# Mind-Body Connection: Impact of Self-Reported Mental Health on Chronic Disease in Older Women (NSHAP Waves 2 and 3)

**DOI:** 10.1093/geroni/igaf122.3219

**Published:** 2025-12-31

**Authors:** Annelie Persson, Julie Blaskewicz Boron

**Affiliations:** University of Nebraska Omaha, Omaha, Nebraska, United States; University of Nebraska Omaha, Omaha, Nebraska, United States

## Abstract

Women experience significant hormonal changes during menopause, which can affect both mental health and the risk of chronic diseases. Although the relationship between subjective and objective mental health is not well understood, subjective cognitive and emotional health has been linked with cognitive decline and chronic disease outcomes. This raises the question of whether self-reported mental health (SRMH) might serve as a valuable metric for predicting chronic disease outcomes among post-menopausal women. The current study investigated SRMH as a predictor of chronic disease outcomes, including heart conditions, non-skin cancers, diabetes, and dementia/mild cognitive impairment (MCI). Participants included 578 women (Mean age: 70.5; Range:62-89) from the National Social Life, Health, and Aging Project who participated during waves 2 (2010-2011) and 3 (2015-2016), and were free of chronic disease diagnoses at wave 2. Emotional/mental health was rated on a scale from poor to excellent (Wave2), and modeled as a predictor for chronic disease outcomes (Wave3). Logistic regression analyses showed relationships between SRMH (Wave2) and chronic disease outcomes five years later for dementia/MCI (p=.02, SE: 0.40, CI: -1.78 to -0.18, OR:0.39), heart conditions (p = 0.0054, SE: 0.182, CI: 0.159 to 0.874, OR: 1.66), and a trend for cancers other than skin (p = 0.087, SE:0.23, CI: -0.834 to 0.0582, OR:0.68). Findings suggest that improving mental health may lower cancer risk and reduce dementia/MCI in older women. The unexpected positive relationship with heart conditions warrants further investigation. Future research should explore variables that might mediate/modify these relationships, such as lifestyle factors or social support systems.

